# Baltimore College of Dental Surgery

**Published:** 1890-04

**Authors:** 


### Editorial, Etc.

BALTIMORE COLLEGE OF DENTAL SURGERY.—The fiftieth annual commencement exercises of the Baltimore College of Dental Surgery took place at Ford's Opera-House, Baltimore, Md., on Thursday, March 20, 1890, at 2 P. M.

The annual oration was delivered by Rev. S. D. Noyes, and the valedictory by U. V. Withee, D. D. S., of the graduating class. The number of matriculates for the session was one hundred and fifty nine.

The degree of D. D. S. was conferred on the following graduates by Prof. R. B. Winder, dean of the faculty.

| NAME. | STATE OR COUNTRY. |
|---|---|
| J. B. Archer | California |
| J. P. Bayon | Louisiana |
| T. A. Beal | Pennsylvania |
| A. L. Beard | Wisconsin |

| | |
|---|---|
| J. Noyes Beemer - - - - | New York |
| J. H Benton, M. D. - - - | N. Carolina |
| H. J. Burkhart - - - - | New York |
| J. Clarence Busey - - - | Maryland |
| A. Foley Butler - - - - | Maryland |
| J. E. Davidson - - - - - | Georgia |
| C. R. Diffenderffer - - - - | Maryland |
| H. A. Donaldson - - - - | Dist. Columbia |
| J. E. Duggar - - - - - - | N. Carolina |
| J. E. Freeland - - - - - | N. Carolina |
| B. D. Friedenwald - - - - | Maryland |
| A. H. Goodwin - - - - | N. Brunswick |
| F. M. Hampton - - - - | Alabama |
| W. M. Hasgrave - - - - | N. Carolina |
| J. H. Harrington - - - - | Kentucky |
| C. A. Hews - - - - - - | Maine |
| W. E. Holt - - - - - | New York |
| M. H. Hunter - - - - - - | N. Carolina |
| W. G. Jankens - - - - - | Virginia |
| L. D. Kelley - - - - - - | Maryland |
| R. L. Lamar - - - - - | Georgia |
| J. H. London - - - - - - | N. Carolina |
| T. M. Lynn - - - - - - - | Iowa |
| W. H. Mathews - - - - | New Jersey |
| H. C. McBrair - - - - | New York |
| F. L. McGraw - - - - | New York |
| W. L. Minson - - - - - | Georgia |
| C. W. Minton - - - - | Pennsylvania |
| W. W. Niles - - - - - | New York |
| J. A. Noll - - - - - | New York |
| H. Ordonez - - - - - - | S. America |
| B. M. Oxley - - - - - - | Nova Scotia |
| J. E. Parker - - - - - - | California |
| J. T. Parker - - - - - | California |
| L. R. Pennington - - - - | Delaware |
| G. A. Potter - - - - - | New York |
| W. S. Pyle - - - - | Maryland |
| F. M. Readio - - - - | Massachusetts |
| R. W. Reese - - - | N. Carolina |

| | |
|---|---|
| E. H. Reed - - - - | Georgia |
| H. N. Richardson - - - | W. Virginia |
| J. E. Rutledge - - - | S. Carolina |
| W. J. Selby - - - | Maryland |
| E. O. Sims - - - - | Florida |
| F. E. Smith - - - | Connecticut |
| D. T. Smithwick - - | N. Carolina |
| A. B. Soule - - - | Vermont |
| W. H. Spangler - - - | Maryland |
| W. M. Spaulding - - - | Minnesota |
| W. Sproul - - - - | Canada |
| H. M. Staire - - - | California |
| R. W. Steuart - - - | Indiana |
| F. C. Street - - - | Maryland |
| A. G. Strickler - - - | Pennsylania |
| J. C. Sutherland - - - | Maryland |
| A. Vietz - - - | Nova Scotia |
| E. H. Webb - - - | S. Carolina |
| E. T. White - - - | New Zealand |
| J. F. Wilson - - - | S. Carolina |
| U. V. Withee - - - | Maine |

